# Neutron reflectometry instrumentation at the ISIS source: current state

**DOI:** 10.1107/S1600576726000397

**Published:** 2026-02-17

**Authors:** Mario Campana, Andrew Caruana, Luke Clifton, Stephen Hall, Arwel Hughes, Christy Kinane, Maximilian Skoda, Oleksandr Tomchuk, John Webster

**Affiliations:** a ISIS Neutron and Muon Source STFC Rutherford Appleton Laboratory DidcotOX11 0QX United Kingdom; Lund University, Sweden; Keele University, United Kingdom

**Keywords:** neutron reflectometry, reflectometers, time of flight, surfaces, interfaces

## Abstract

Neutron reflectometry (NR) at the ISIS Neutron and Muon Source has evolved into a mature, versatile technique for investigating the structure and dynamics of interfaces across a wide range of scientific disciplines, including soft matter, magnetism, quantum materials and environmental systems. This article provides a comprehensive overview of the current instrumentation available at ISIS, detailing the capabilities of the four operational reflectometers – OFFSPEC, INTER, POLREF and SURF – including their time-of-flight configurations, scattering geometries, polarization options and range of accessible sample environments. Special emphasis is placed on the integration of advanced sample environments, automated control software and data analysis tools, including *IBEX*, *MANTID* and the *ISIS Data Analysis as a Service* (*IDAaaS*) platform, which collectively facilitate efficient, reproducible and high-quality NR measurements. Representative scientific highlights demonstrate the unique potential of NR to resolve sub-nanometre structural and kinetic information in biological membranes, thin functional films, quantum fluids and environmental interfaces. By consolidating detailed technical information, operational characteristics and examples of cutting-edge research, this article serves as a practical guide for new and experienced users, helping them design experiments, select suitable instruments and sample environments, and fully exploit the capabilities of ISIS neutron reflectometers.

## Abstract 2

This article presents the current capabilities of the four time-of-flight neutron reflectometers at the ISIS Neutron and Muon Source, highlighting recent advances in instrumentation, automation and data analysis that enable high-precision studies of interfacial structures across diverse scientific fields.

## 1. Introduction

Over nearly half a century since the publication of the first neutron reflectivity curve (Hayter *et al.*, 1976 ▸), neutron reflectometry (NR) has evolved from an exotic experimental technique to a widespread method for the analysis of biological, magnetic, quantum and many other systems. Its isotope and spin sensitivity, combined with the high penetration depth and non-destructive nature of neutrons, makes it uniquely suited to deliver information on the structure and kinetics of layered nanosystems that underpin their functional properties (Rogers *et al.*, 2009 ▸). It is one of a very few techniques capable of measuring buried interfaces in terms of scattering length density (SLD) profile and magnetization (Penfold & Thomas, 1990 ▸; Ankner & Felcher, 1999 ▸).

Interfaces play an essential role in nature, governing many processes. They are equally important in controlling key chemical and biological mechanisms through their catalytic, mechanical, electromagnetic and many other properties. Interfaces are scientifically crucial for trans-cell transport, ferroelectricity and superconductivity. Among available experimental approaches, NR is distinctive in its ability to probe buried interfaces while providing a statistically meaningful description of their properties (Skoda, 2019 ▸; Lakey *et al.*, 2022 ▸; Causer *et al.*, 2023 ▸).

The effectiveness of neutron reflectometry as a non-invasive characterization method has driven the continuous development of instrumentation. According to the Open Reflectometry Standards Organisation (https://www.reflectometry.org), 44 neutron reflectometers are currently in operation worldwide, located at both pulsed and steady-state neutron sources.

The ISIS Neutron and Muon Source is the home of the world’s first dedicated neutron reflectometer, CRISP, which operated successfully for several decades (Penfold *et al.*, 1987 ▸). Today, this beamline serves as an internal test facility for the further development of neutron scattering technologies, including detector and sample environment projects.

In addition to CRISP, there are another four high-flux neutron reflectometers across ISIS: OFFSPEC, POLREF, INTER and SURF. These instruments form part of the ISIS user programme and support a comprehensive range of scientific applications. They provide access to surfaces and buried interfaces, as well as free liquid surfaces, and enable the study of magnetic materials. The unique properties of neutrons allow for the possibility to perform measurements in realistic sample environments (Sivia, 2011 ▸). External variables such as temperature, pressure or magnetic field can all be applied to a variety of interfaces between different states of matter.

The present article reports on the status of the ISIS reflectometers. It describes components of the NR instruments after recent upgrades, lists the control software and sample environments available, and provides an overview of recent achievements in line with the ISIS scientific strategy.

## 2. The neutron source

ISIS is one of the world’s leading pulsed neutron and muon facilities, located at the STFC Rutherford Appleton Laboratory in the UK (Thomason, 2019 ▸). It generates short, bright pulses of neutrons by accelerating protons to 800 MeV and directing them onto a heavy-metal spallation target. The acceleration chain consists of a linear accelerator and a 163 m synchrotron ring. Negative hydrogen ions (H^−^) are produced, bunched and accelerated in the linac before entering the synchrotron, where the electrons are stripped, leaving protons. After roughly 10^4^ revolutions, the proton bunches reach 84% of the speed of light and are extracted by fast kicker magnets.

The proton bunches strike a tungsten target, releasing neutrons in a process known as spallation. The resulting neutron pulse is both intense and short lived, allowing time-of-flight (TOF) measurements with high resolution. The neutrons are moderated to thermal and/or cold energies by moderators surrounding the target. The broad polychromatic spectrum of neutrons is then analysed by measuring their flight times to the detectors, avoiding the need for monochromation and the associated loss of neutrons. The scattering vector components, 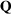, are typically scanned by varying the neutron wavelength, recalculated from the TOF.

ISIS has two target stations (TS1 and TS2) operating at 50 Hz with every fifth pulse sent to TS2. TS1, operational since 1984, supports a wide range of instruments across physics, chemistry, materials science and engineering. TS2, commissioned in 2009, was specifically optimized for experiments that benefit from longer wavelengths and high flux at small momentum transfer, such as soft-matter and surface studies (Bennington, 2009 ▸). TS2 runs at 10 Hz, giving a 100 ms TOF window and enabling measurements of wavelengths up to 20 Å at good resolution with moderate instrument lengths. Liquid hydrogen moderators of special design are used for neutron reflectometry, maximizing flux into the instruments, especially at longer wavelengths (Picton *et al.*, 2005 ▸). Viewing the lower liquid hydrogen moderator on TS1, SURF provides a high-flux polychromatic neutron beam suitable for rapid measurements. Operating on TS2, OFFSPEC, INTER and POLREF use a common grooved H_2_ moderator.

Neutron beams are transported from the moderators to the instrument suite. For SURF at TS1 the beam is guided down a neutron-absorbing shielded collimation tube (Penfold *et al.*, 1997 ▸). For INTER, OFFSPEC and POLREF at TS2 the beam passes through evacuated 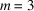 neutron guides (Webster *et al.*, 2011 ▸; Charlton *et al.*, 2011 ▸). To minimize potential fast neutron background a multichannel supermirror bender was additionally installed on OFFSPEC (Dalgliesh *et al.*, 2011 ▸). The ISIS reflectometers’ performance and scientific applications are described in the following sections.

## 3. The instrument suite

### 3.1. Principal schemes and current characteristics of the reflectometers

**Figure 1:**
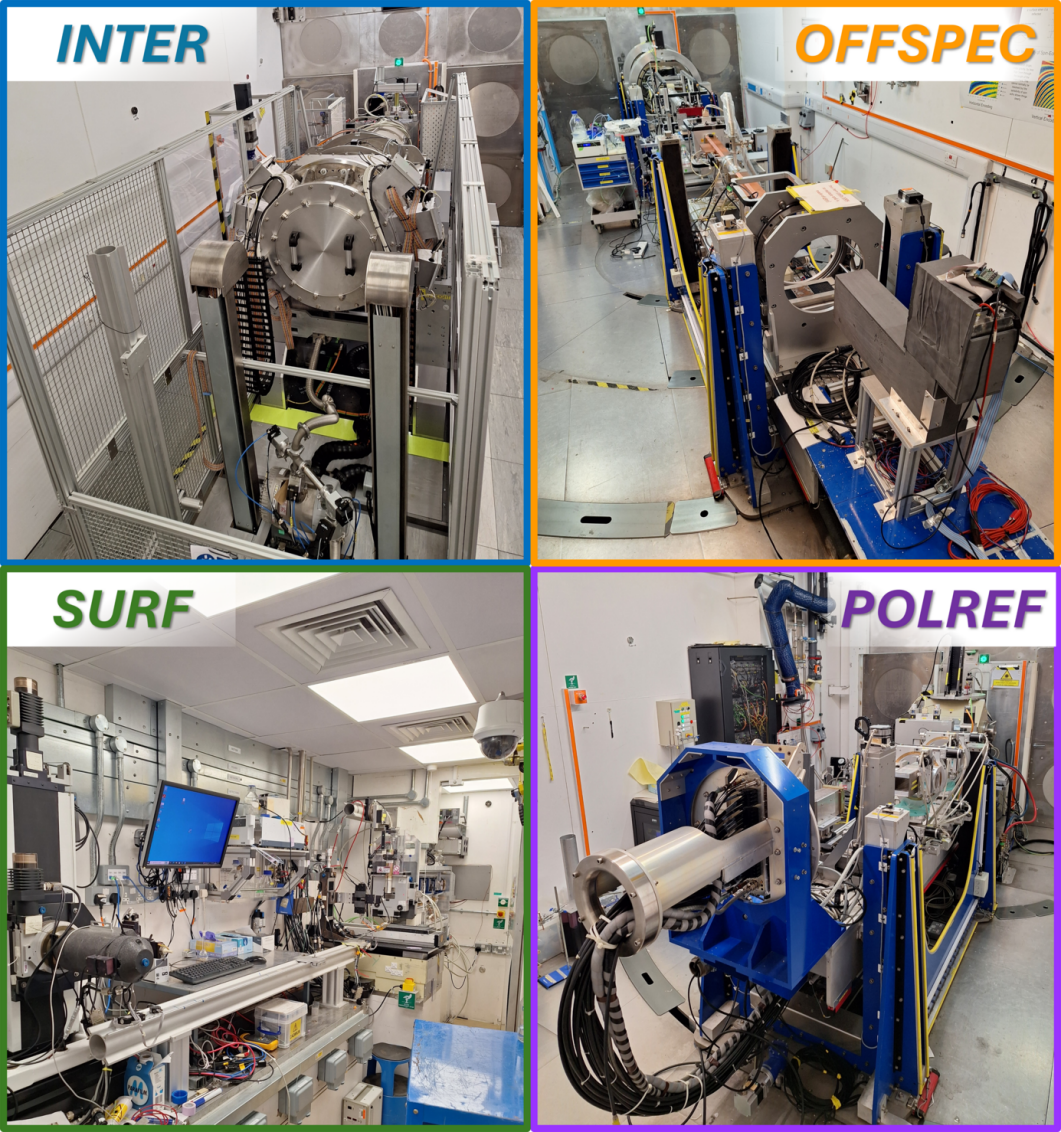
Sample–detector areas of ISIS reflectometers after recent upgrades.

**Figure 2:**
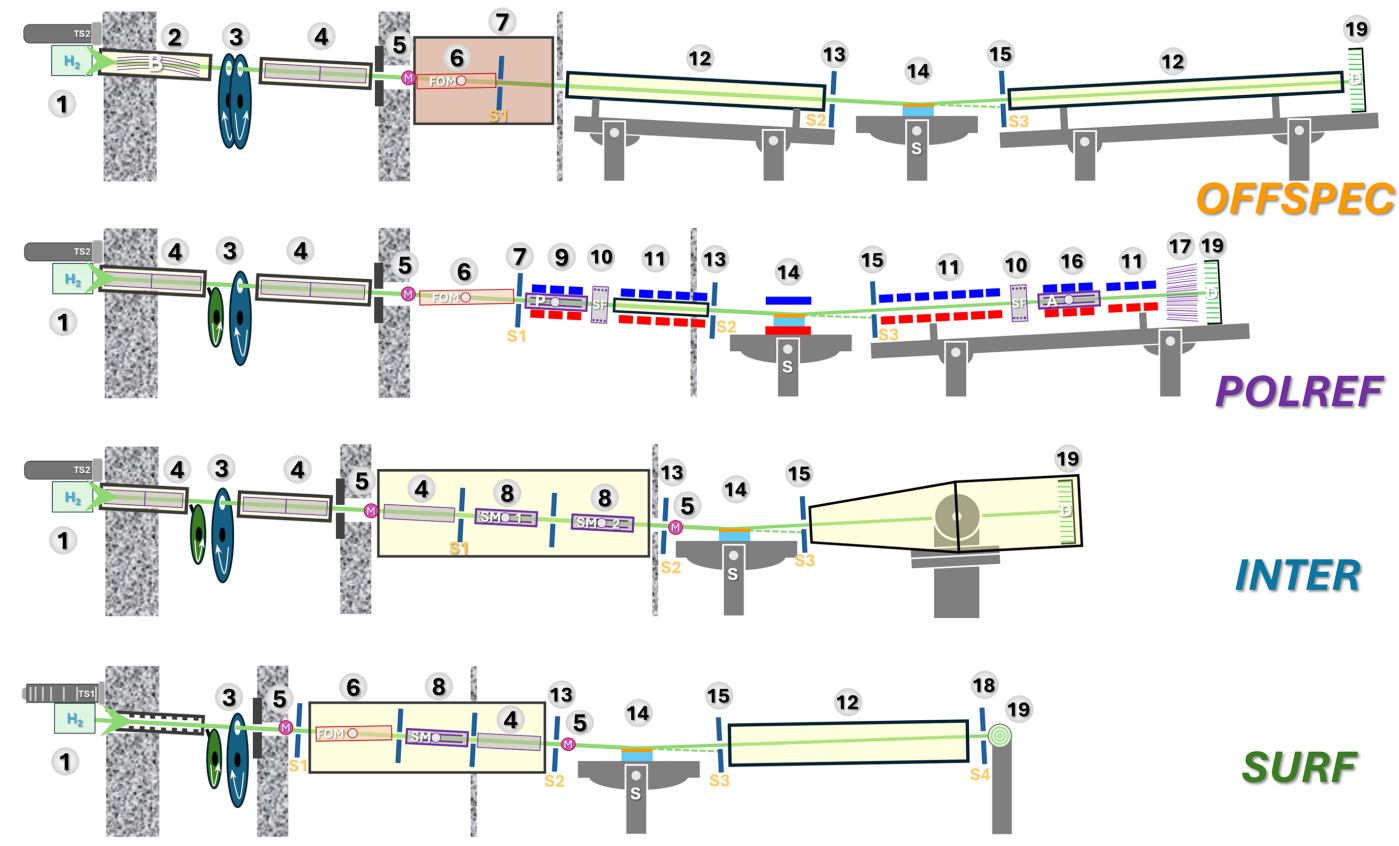
Sketch representation of the reflectometry suite. 1 – target/moderator; 2 – bender; 3 – chopper system; 4 – neutron guide; 5 – monitor detector; 6 – frame overlapping mirror; 7 – first collimation slit (slit 1); 8 – supermirror; 9 – polarizer; 10 – spin-flipper; 11 – magnetic guiding field; 12 – flight tube; 13 – pre-sample slit (slit 2); 14 – five-axis sample position (three translations plus two rotations) equipped with anti-vibration table; 15 – post-sample slit (slit 3); 16 – specular analyser; 17 – off-specular analyser; 18 – pre-detector slit (slit 4); 19 – detector. Units with a yellow background are under vacuum; OFFSPEC cloche – an enclosure for the pre-sample optics – is filled with helium for background suppression.

The ISIS Neutron and Muon Source hosts a state-of-the-art suite of four TOF neutron reflectometers (Fig. 1 ▸), providing world-class capabilities for the study of surfaces and interfaces across a broad range of scientific disciplines. Together, SURF, INTER, POLREF and OFFSPEC cover both horizontal and vertical scattering geometries, support polarized and unpolarized measurements, and offer flexible resolution settings for specular, off-specular and grazing-incidence small-angle scattering studies (see principal principle diagrams in Fig. 2 ▸). The instruments deliver high-flux pulsed neutron beams with wavelength coverage from 0.5 Å up to 17 Å. A wide range of sample environments are available across the suite, enabling experiments under controlled temperature, magnetic field and electrochemical conditions, as well as studies of free liquid surfaces and buried multilayers. This combination ensures that the ISIS reflectometers can address the full spectrum of scientific problems in soft matter, magnetism, quantum materials and advanced functional interfaces.

SURF is a horizontal-plane neutron reflectometer designed for the study of free liquid surfaces and buried interfaces (Table 1 ▸) (Penfold *et al.*, 1997 ▸). SURF operates over a wavelength band of roughly 0.5–7 Å and supports grazing angles up to 5°, covering a wide *Q_z_* range. The installation of a new evacuated cloche, coupled with an update to the mechanics of the collimation system and the installation of focusing guides, made it possible to reduce the incoherent background. An incident beam inclined at 1.5° below the horizontal allows, with the use of a non-polarizing supermirror, the measurement of liquid surfaces. SURF’s fixed-sample geometry and low-background design make it well suited for studying capillary-wave roughness, adsorption at fluid interfaces and kinetic processes at the air–liquid interface (Table 2 ▸).

INTER is a versatile high-intensity horizontal-plane reflec­tometer optimized for both solid and liquid interfaces (Webster *et al.*, 2006 ▸). The installation of focusing guides increased the neutron flux by a factor of about 4. The presence of two supermirrors provides additional variability in angles relative to the sample surface, allowing the beam to bounce both up and down at the sample point. INTER supports a wide range of sample environments, from temperature-controlled Langmuir troughs to magnetic and electrochemical cells, making it highly suitable for soft-matter, biological and electrochemical interface studies.

POLREF is a polarized TOF neutron reflectometer designed for the investigation of magnetic and non-magnetic layered structures, primarily for hard condensed matter (Webster *et al.*, 2011 ▸). POLREF is optimized for rapid high-contrast measurements with full polarization and analysis capability, enabling the separation of nuclear and magnetic scattering contributions in polarized neutron reflectometry (PNR) and polarization analysis (PA) measurements, both specular and off-specular. It supports a wide range of sample environments including cryogenic, magnetic and high-temperature setups with *in situ* transport, making it a key instrument for magnetic thin film and spintronic research.

OFFSPEC is a low-background time-of-flight neutron reflectometer optimized for the study of surfaces and interfaces, with a particular strength in measuring off-specular scattering (Webster *et al.*, 2011 ▸; Dalgliesh *et al.*, 2011 ▸). Having a multi-slit optical beam diverter (bender) and viewing the same grooved hydrogen moderator of TS2 as INTER and POLREF, it provides access to nanometre length scales both parallel and perpendicular to interfaces. OFFSPEC’s position-sensitive detector allows simultaneous collection of specular and diffuse scattering, making it ideal for total off-specular reflectometry. The instrument supports a wide range of sample environments, enabling applications across biophysics, soft matter and materials science.

The ISIS neutron reflectometers, while versatile, are each optimized to address specific measurement approaches (Table 2 ▸). Thus, all experiments requiring polarization, magnetic contrast, high magnetic fields or ultra-low temperatures can be carried out on POLREF. The focusing optics of INTER enable the study of small samples or rapid kinetics. SURF, originally designed as a liquid reflectometer, is particularly well suited for investigations into the physical chemistry of liquid systems. A distinctive feature of OFFSPEC is its long flight path, non-direct source view and extended sample-to-detector distance, which together provide enhanced resolution and low background.

**Figure 3:**
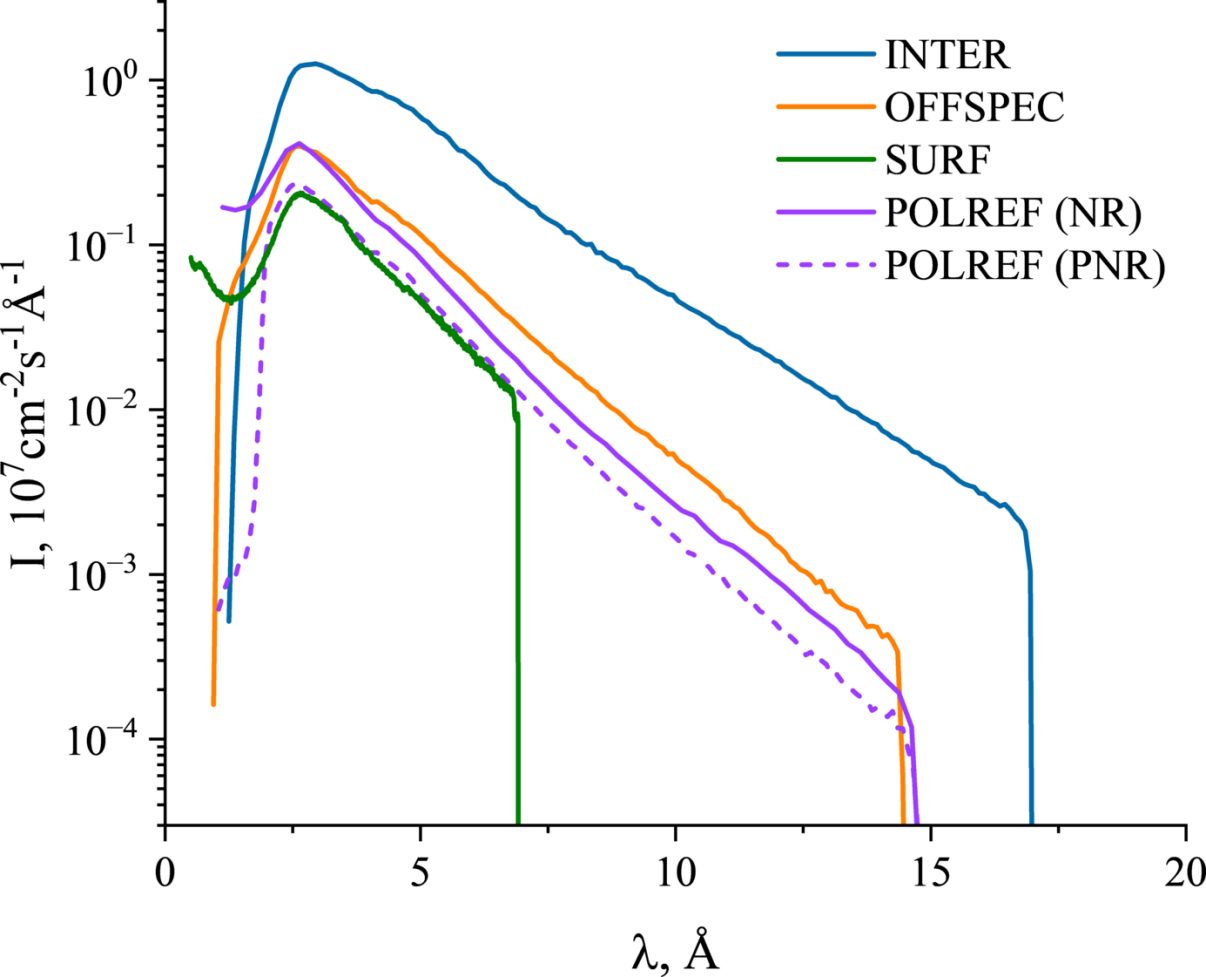
Comparison of TOF spectra of the neutron beam at the sample position for ISIS reflectometers. The integral flux characteristics are shown in Table 3 ▸.

Each beamline is equipped with a monitor detector for correction for detector efficiency, source power and spectral shape. OFFSPEC, INTER and POLREF are equipped with position-sensitive detectors (Table 1 ▸). In the case of POLREF it is a microstrip gas chamber OSMOND detector (Bateman *et al.*, 2013 ▸). OFFSPEC has a linear scintillator of wavelength-shifting fibre type (Khaplanov *et al.*, 2025 ▸). INTER employs a modular detector plate made of eight sections of scintillator with wavelength-shifting optical fibres, which allows simultaneous acquisition of off-specular scattering (Richardson *et al.*, 1997 ▸; Pynn *et al.*, 1999 ▸). Combined with multi-axis sample stages and focusing optics, this enables measurements in the π-GISANS mode (Vorobiev *et al.*, 2021 ▸) – an implementation of grazing-incidence small-angle neutron scattering (GISANS) (Müller-Buschbaum *et al.*, 2004 ▸; Köhler *et al.*, 2026 ▸) with reduced resolution in one direction. The quasi-two-dimensional design of the INTER detector allows test measurements by conventional GISANS with low *Q_y_* resolution in the standard horizontal sample geometry. Currently,SURF is equipped with a helium tube, but further upgrades will include the installation of a one-dimensional position-sensitive detector. A comparison of the TOF spectra at the sample position is shown in Fig. 3 ▸. Overall, relatively high neutron fluxes over a wide wavelength band are observed for all instruments, but the INTER focusing optics together with high TS2 efficiency and short flight path allow higher values compared with other considered beamlines.

When experiments require illumination of liquid–liquid or liquid–gas interfaces from below the horizontal plane, INTER with its pair of supermirrors should be considered the first choice. Standard experiments on free liquid surfaces can be performed on any of the reflectometers, since these beamlines are initially inclined relative to the horizontal, but such experiments are typically scheduled for SURF or INTER.

### 3.2. Sample environment

Many features of the instrument sample positions are common, and most sample environment equipment can be deployed with equal ease on each beamline. The principal components of each sample position are two flexible crossed goniometers, two vertical translation stages, horizontal translation stages and an active anti-vibration control unit. The centre of rotation of the transverse goniometer is 450 mm above the sample table, which is sufficient for the mounting of most sample environments (Table 3 ▸). At the sample position, there is approximately 1000 mm of free space along the beam. A sample changer is not required, since there is a horizontal translation across the beam of about 600 mm. The footprint is adjusted by slits in the collimation system while maintaining the specified resolution. Pre-alignment is achieved using a pre-installed laser that follows the path of the neutrons.

Precise control of experimental conditions is critical for neutron reflectometry, necessitating dedicated sample environments. In soft-matter experiments, the neutron beam is typically highly collimated and interacts with relatively large sample surfaces, making the structural properties highly sensitive to variations in experimental parameters such as temperature, humidity *etc*. For studies at air–liquid interfaces, sample areas may reach large area values (80–200 cm^2^), allowing minimization of meniscus effects, whereas for solid–liquid interfaces the illuminated footprint must generally be more restricted (20–30 cm^2^) to ensure measurement accuracy. For hard condensed matter, samples are often small in area (down to 0.1 cm^2^), requiring long count times. This necessitates very stable magnetic fields and temperatures to allow long data collection times.

Table 3 ▸ summarizes the range of sample environments currently available for NR investigations of both soft and hard condensed matter, highlighting their capabilities and operational constraints. To provide a comprehensive overview, the available capabilities for sample preparation and in-line char­acterization are also considered. This list allows for almost any neutron reflectometric experiment to be performed, and ex­per­imental setups customized for users’ requirements can be quickly adapted with the support of instrument scientists and the Soft Matter Team of the ISIS Sample Environment Group.

### 3.3. Related software

ISIS neutron reflectometry experiments are controlled by the *IBEX* environment (Akeroyd *et al.*, 2021 ▸). *IBEX* is a modern, *EPICS*-based (Dalesio *et al.*, 1994 ▸) software framework developed at the ISIS Neutron and Muon Source to control and coordinate neutron and muon scattering experiments, replacing the previous control system. It provides a distributed architecture for loosely coupled client and server applications, with server components handling device control and business logic, and clients using *Control System Studio* (Eclipse/RCP) (https://www.controlsystemstudio.org/) for user interfaces. Key functions of *IBEX* include the *Instrument Control Program* (*ICP*) for managing data acquisition and the *IBEX* server for coordinating various experimental aspects.

Data reduction is done via *MANTID* (Arnold *et al.*, 2014 ▸). *MANTID* is an open-source software framework, co-developed by major neutron scattering facilities, for the reduction and analysis of neutron and muon scattering data. It provides a graphical user interface and a Python API to convert raw time-of-flight data into instrument-independent quantities like the reflectivity. The software handles various data corrections (*e.g.* divergent beam or non-planarity), performs data reduction, and offers tools for visualization and detailed analysis for different scattering techniques including NR.

*ISIS Data Analysis as a Service* (*IDAaaS*), built on the Ada platform (https://ada.stfc.ac.uk/), offers a virtual computing environment for the reduction and analysis of neutron scattering data. This service is maintained and supported by the ISIS Neutron and Muon Source, allowing users to access dedicated computing resources without the need for local installations. *IDAaaS* facilitates standardized workflows for data processing, ensures compatibility with instrument-specific software and provides a secure environment for collaborative work. By leveraging this virtual infrastructure, users can efficiently perform data reduction, fitting and modelling of re­flec­to­metry, streamlining the transition from raw neutron counts to scientifically interpretable results. The *IDAaaS* environment comes equipped with a range of preinstalled fitting and modelling tools specifically tailored for neutron reflectometry, enabling users to perform layer-profile analysis and extract structural parameters directly within the virtual platform.

## 4. Selected scientific highlights

**Figure 4:**
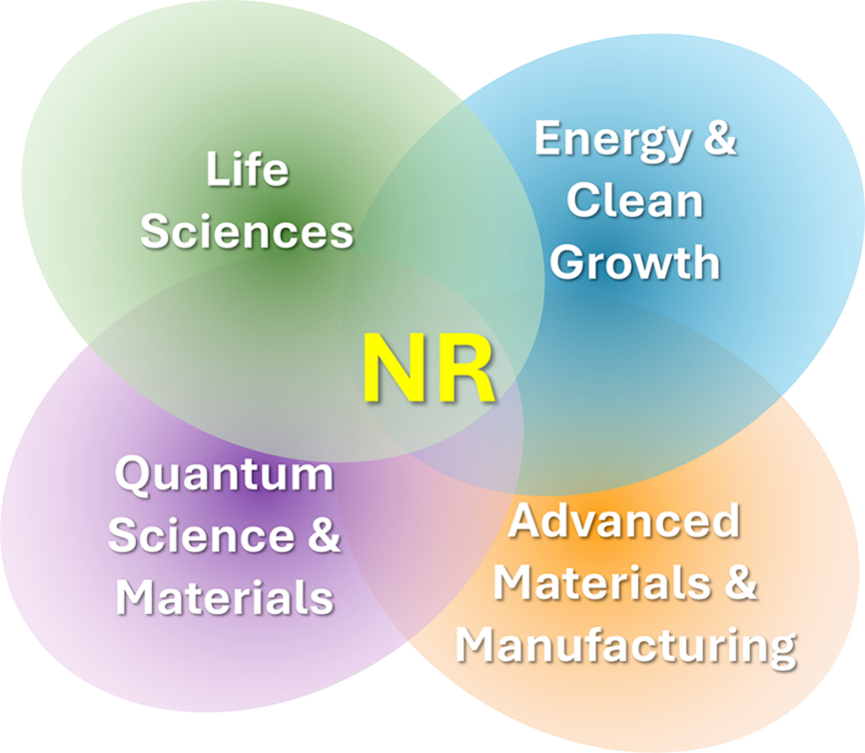
Focused scientific areas at the ISIS source.

A wide variety of science is carried out using the beamlines at ISIS Neutron and Muon Source. According to the current ISIS strategy, priority is given to four scientific areas, which can be conditionally designated as life sciences, advanced materials and manufacturing, quantum science and materials, and energy and clean growth (Fig. 4 ▸). Experiments on these topics are widely represented across NR instruments. Below are examples of research conducted in these areas where neutron reflectometry played a key role.

NR at ISIS has played a pivotal role in advancing life sciences research, particularly in understanding biomolecular interactions at membranes and interfaces with nanometre precision. The recent study of Maset *et al.* (2025 ▸) unravelled the mechanisms of action of synthetic nanoengineered antimicrobial polymers, revealing how they disrupt Gram-negative bacterial envelopes through lipopolysaccharide targeting, pore formation and membrane dissolution. NR has also provided unique structural information on human immunodeficiency virus fusion peptide interactions with model membranes, showing how membrane phase state, charge distribution and compressibility govern peptide penetration and host membrane dehydration, with implications for viral entry inhibition (Swarnakar *et al.*, 2025 ▸). Complementary investigations combining NR with microbiological assays have demonstrated the synergistic enhancement of antibiotic efficacy when paired with designed lipopeptides, directly correlating molecular-scale membrane disruption with improved uptake and rapid bacterial killing, including against antimicrobial-resistant strains (Liao *et al.*, 2025 ▸). Together, these examples highlight how NR enables direct, quantitative observation of structural and compositional changes in complex biological membranes, offering a crucial experimental foundation for the development of novel antimicrobial therapies and antiviral strategies.

**Figure 5:**
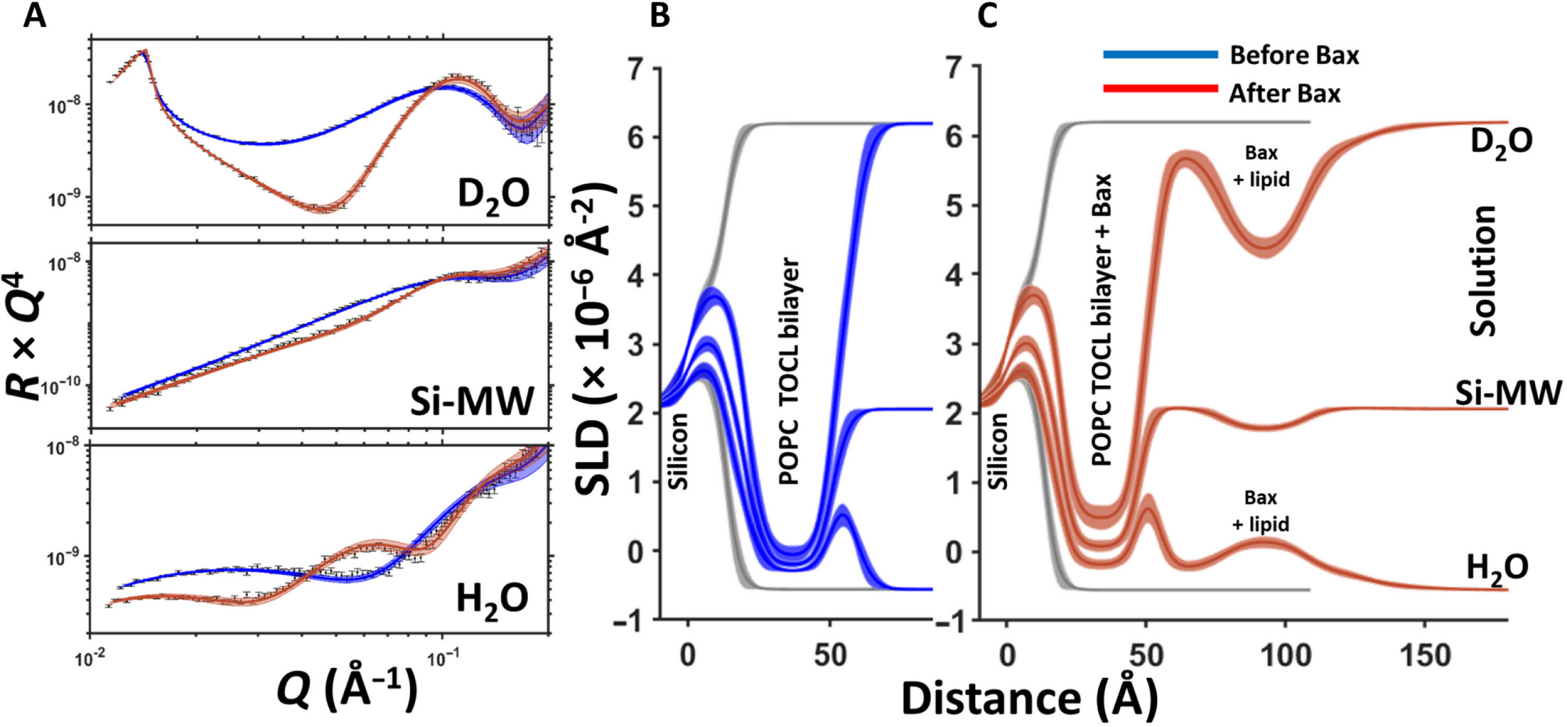
NR data (error bars) and model data fits (lines) from a 90 mol% 1-palmitoyl-2-oleoyl-phosphocholine (POPC)–10 mol% tetraoleoyl cardiolipin (TOCL) supported lipid bilayer before (blue) and after (red) the interaction of natural abundance hydrogen h-Bax are shown in three differing solution isotopic contrast conditions, being D_2_O, Si-MW and H_2_O (A) buffer solutions. The SLD profiles and the model fits are shown for the surface structure before (B) and after (C) the h-Bax interaction. Reproduced from Clifton *et al.* (2023 ▸). Licenced under CC BY 4.0.

Work on programmed cell death (Clifton *et al.*, 2023 ▸) used SURF and OFFSPEC instruments to exploit NR’s ability to probe buried, fully hydrated interfaces with sub-nanometre resolution under near-physiological conditions, providing statistically robust structural and kinetic insights. Apoptosis is a fundamental process in development and tissue homeostasis, with dysregulation linked to neurodegeneration and cancer. A key step is the permeabilization of the mitochondrial outer membrane by the Bcl-2 family protein Bax, but its molecular mechanism has been debated. Using model membranes mimicking mitochondrial composition, the authors resolved two distinct kinetic phases: rapid Bax adsorption within minutes, followed by a slower process where Bax extracts lipids and assembles Bax–lipid clusters on the membrane surface. Cluster formation coincided with pore opening, establishing a causal link between lipid extraction, cluster assembly and membrane permeabilization. Crucially, cardiolipin content was shown to accelerate adsorption and enhance pore formation, demonstrating that mitochondrial lipids actively participate in pore creation rather than serving merely as a passive matrix. NR, combined with complementary spectroscopies, thus delivered a detailed mechanistic picture of Bax-mediated membrane permeabilization and offers a framework for studying other membrane-active proteins relevant to health and disease (Fig. 5 ▸).

The advanced materials field is represented by several research directions, including hydrogen technology. The transition to a hydrogen-based energy economy requires safe, accurate and cost-effective hydrogen sensors, and NR has emerged as a powerful tool to optimize the materials underpinning such devices. Recent studies on thin-film Ta-based metal hydrides, carried out at OFFSPEC, have demonstrated that NR, when combined with *in situ* hydrogen loading, provides quantitative depth profiles of hydrogen concentration and layer expansion, revealing how alloying can be used to systematically tune the sensor’s operating window. For example, in Ta–Ru alloys, the formation of a homogeneous solid solution up to ∼30 at.% Ru was confirmed, and NR showed that increasing Ru content compresses the lattice, reduces hydrogen uptake and shifts the hydrogenation enthalpy – allowing the pressure/concentration range of optical hydrogen sensors to be tuned over more than seven orders of magnitude in pressure while maintaining hysteresis-free response (Bannenberg *et al.*, 2023 ▸).

**Figure 6:**
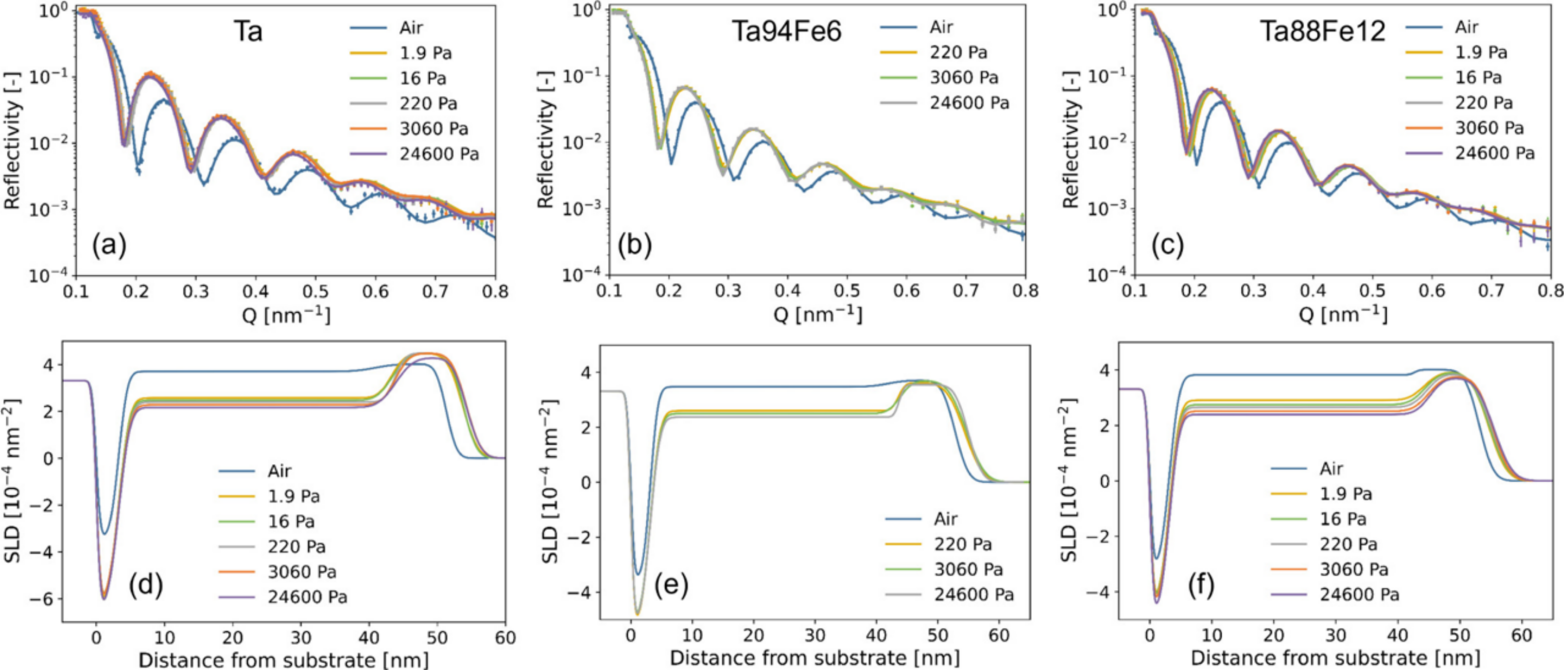
*In situ* NR results of 40 nm Ta_1−*y*_Fe_*y*_ thin films with a 4 nm Ti adhesion layer and capped with 10 nm of Pd_0.6_Au_0.35_Cu_0.05_ at *T* = 22°C. (*a*–*c*) Reflectograms of the Ta_1−*y*_Fe_*y*_ thin films with (*a*) *y *= 0, (*b*) *y* = 0.06 and (*c*) *y* = 0.12 measured for the hydrogen pressures indicated in the legend and for increasing pressure steps. The continuous lines represent the fits of a model to the data. SLD profiles for (*d*) *y* = 0, (*e*) *y* = 0.06 and (*f*) *y* = 0.12. Reproduced from Bannenberg *et al.* (2024 ▸). Licenced under CC BY 4.0.

Complementary work on Ta alloyed with Fe, Co and Ni shows a more complex behaviour, where phase segregation competes with hydrogen uptake (Bannenberg *et al.*, 2024 ▸). NR data revealed only a moderate reduction in absorbed hydrogen with these alloyants, confirming that tuning via Fe/Co/Ni alloying is less effective than Ru substitution, although the resulting thin films still exhibit a reversible and stable optical response to hydrogen exposure across a wide pressure range (Fig. 6 ▸). This highlights the unique capability of NR to directly quantify hydrogen content within buried layers – a key advantage over X-ray-based techniques, which are largely insensitive to light elements such as hydrogen. By combining NR with optical transmission measurements, the authors correlated structural evolution with functional performance: films retaining a body-centred cubic phase showed gradual, reversible hydrogenation behaviour across more than seven orders of magnitude in hydrogen pressure, whereas samples dominated by the amorphous phase exhibited negligible optical response. Together, these studies underscore NR’s unique role in directly probing hydrogen distributions in functional thin films, thereby guiding the rational design of high-performance sensing layers and accelerating the development of scalable, manufacturable solutions for the hydrogen economy.

NR and PNR are proving indispensable for disentangling the complex interfacial phenomena that underpin many emerging quantum-materials and spintronic technologies. By delivering depth-resolved maps of both nuclear and magnetic scattering length density with sub-nanometre resolution, NR can directly quantify changes in magnetization, exchange bias and proximity-induced magnetic moments at buried interfaces, and track these properties under applied fields, currents or ionic motion. This capability is central to recent advances: for example, NR/PNR can resolve vacancy/ion-driven magnetic phase changes and associated exchange-bias modulation in magneto-ionic heterostructures (all-Mn-nitride systems), thereby linking nitro­gen transport to reversible ferrimagnetic–antiferromagnetic transitions and large shifts in exchange bias (Chen *et al.*, 2025 ▸). Reflectometry likewise provides the depth-profile information needed to understand how composition modulation and proximity effects in amorphous multilayers tune static magnetization and dynamical damping (Tryggvason *et al.*, 2025 ▸). In spin-orbit-torque and antiferromagnetic switching studies NR can validate interfacial coupling and readout schemes by quantifying spin-polarization transfer and exchange coupling to adjacent ferromagnets (He *et al.*, 2024 ▸). For correlated oxide heterostructures NR/PNR uniquely links strain- and stacking-induced orbital reconstruction to emergent interfacial ferromagnetism and exchange coupling (LNO/LSMO systems), providing the experimental constraints required to test first-principles predictions (Bhatt *et al.*, 2024 ▸). Together, these examples illustrate how PNR furnishes the precise, model-constraining depth information that is essential for designing and optimizing next-generation quantum and spintronic devices. The POLREF polarized reflectometer is now a routine high-sensitivity probe for depth-resolved studies of quantum and magnetic materials, delivering sub-nanometre structural and magnetization profiles under realistic *operando* conditions.

**Figure 7:**
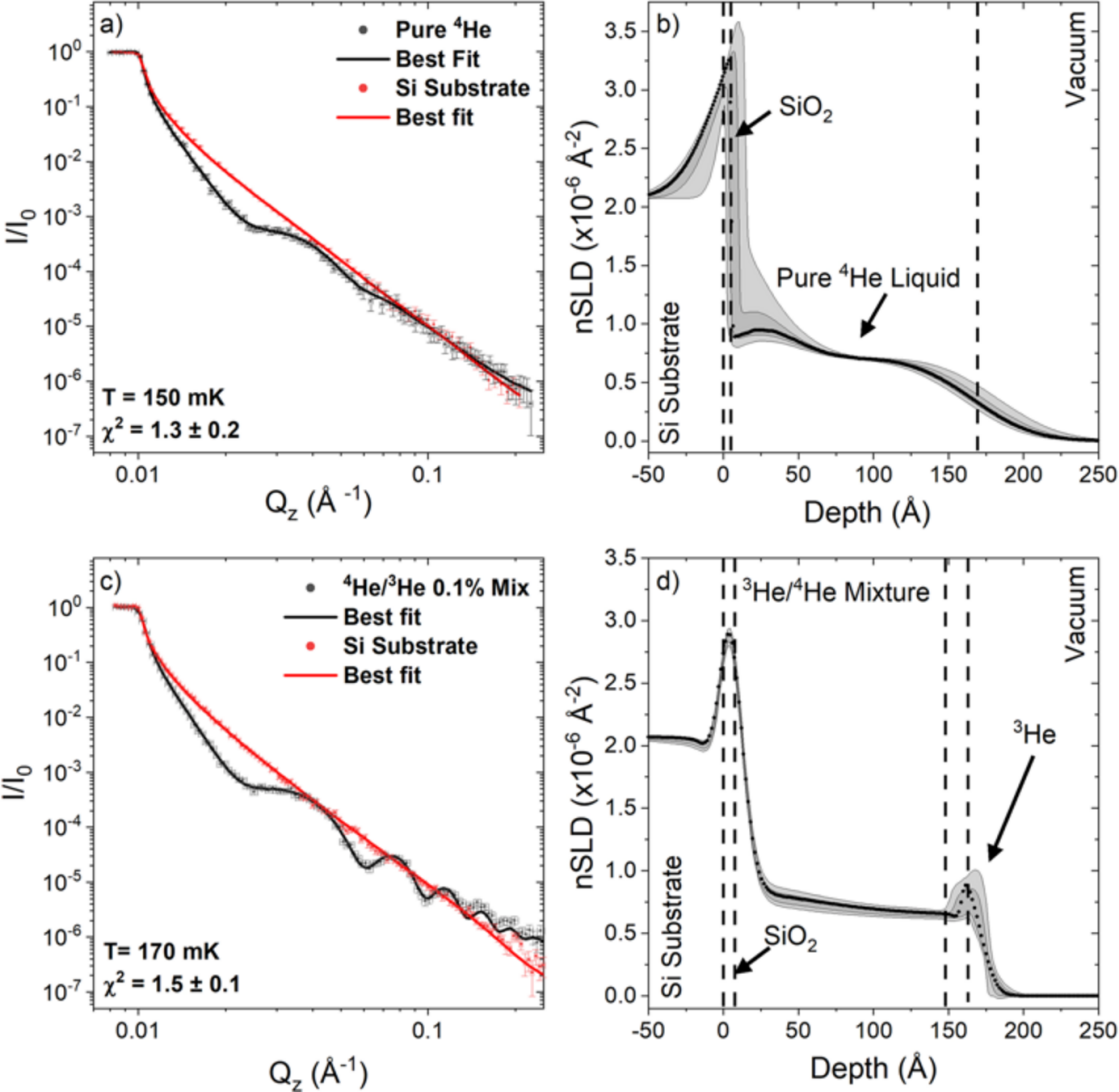
Reflectivity curves and neutron scattering length density profiles for nominally pure ^4^He and for the ^3^He–^4^He mixture. Reproduced from Kirichek *et al.* (2024 ▸). Licenced under CC BY 4.0.

A recent study by Kirichek *et al.* (2024 ▸) demonstrates the unique capability of neutron reflectometry for investigating quantum fluids and nanoscale phase behaviour at millikelvin temperatures. Using the POLREF reflectometer, the authors measured the depth profile of ^3^He in a phase-separated superfluid ^3^He–^4^He film supported on a polished silicon substrate (Fig. 7 ▸). The experiment employed a cryogen-free dilution refrigerator and a custom sample cell, enabling measurements down to ∼170 mK under vibration-minimized conditions. Neutron reflectivity, exploiting the strong absorption contrast of ^3^He, was used to resolve the distribution of ^3^He within the nanometre-thick film with sub-nanometre precision. The results reveal a temperature-dependent reorganization of the film: at the lowest temperatures, a distinct ^3^He-rich surface layer is observed, which gradually dissolves into the underlying ^4^He layer on warming to ∼300 mK, indicating the disappearance of phase separation. Surprisingly, at higher temperatures approaching 1.5 K, the layered structure re-emerges, suggesting a re-entrant phase behaviour. The combination of specular reflectivity and off-specular scattering provided both vertical concentration profiles and information on in-plane correlations, yielding a comprehensive picture of the interfacial structure and its evolution. This work highlights the power of time-of-flight neutron reflectometry to non-invasively probe quantum condensates and isotope-specific layering phenomena under extreme cryogenic conditions. Such experiments expand the scientific reach of reflectometry beyond soft matter and magnetism into the domain of strongly correlated quantum systems, demonstrating its value for exploring exotic states of matter.

**Figure 8:**
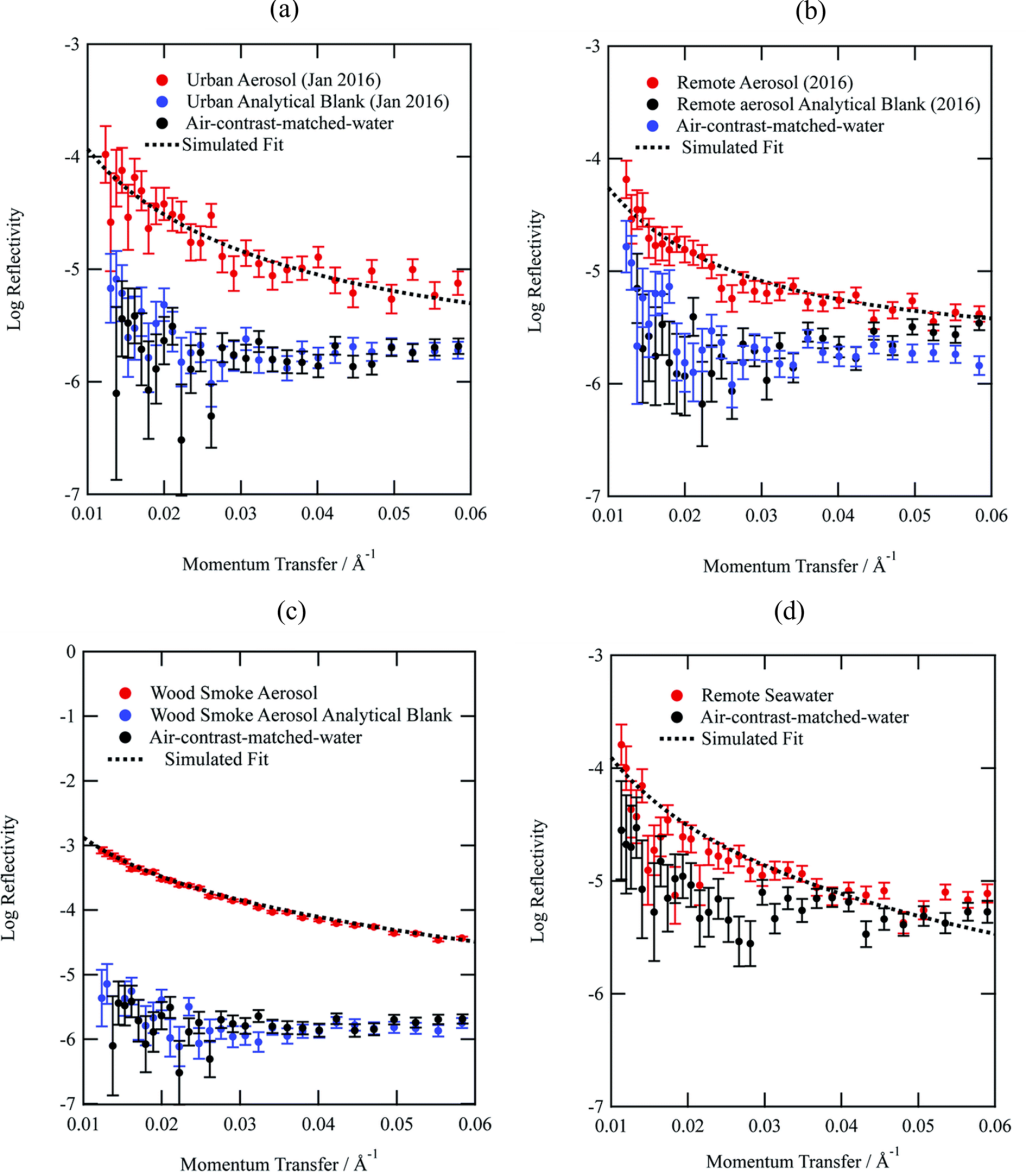
Neutron reflectivity profiles for organic films extracted from (*a*) urban, (*b*) remote, (*c*) wood smoke and (*d*) remote seawater on an air–water interface (red). Also included are the neutron reflectivity profiles for a bare interface (black) and the analytical filter blanks (blue). The figure demonstrates that the analytical blanks are indistinguishable from the bare air–water interface and the samples extracted from the aerosol filter are clearly distinguishable from the bare interface and analytical blanks. The remote seawater sample and bare surface (*d*) were recorded separately from the aerosol samples and their corresponding bare surfaces (*a*–*c*). Reproduced from Shepherd *et al.* (2022 ▸). Licenced under CC BY 3.0

NR is also proving transformative for environment studies, particularly for understanding interfacial processes in atmospheric chemistry. Shepherd *et al.* (2022 ▸) employed NR at INTER to investigate the oxidation kinetics and structural evolution of thin organic films formed at the air–water interface, using materials extracted from atmospheric aerosols of urban, remote (Antarctica) and wood-smoke origins. The films, typically 6–18 Å thick, were exposed to gas-phase hydroxyl (OH) radicals, and their thickness and neutron scattering length density were monitored *in situ*. The results reveal that these organic films are reactive to OH radicals, with oxidation lifetimes on the order of hours, comparable to atmospheric aerosol residence times. This finding underscores the importance of considering film oxidation in atmospheric models. In contrast, the commonly used lipid 1,2-distearoyl-*sn*-*glycero*-3-phospho­choline monolayer exhibited significantly different oxidation behaviour, highlighting the necessity of using real atmospheric materials for such studies. The study demonstrates that NR is an excellent technique for studying thin films at the air–water interface formed from atmospheric aerosol materials, providing insights into their chemical reactivity and structural properties (Fig. 8 ▸).

Investigations of organic films at the air–water interface show that urban and woodland aerosol extracts form ∼0.6 nm interfacial layers that remain largely intact upon SO_2_ exposure, while wood-smoke extracts form thick (> 40 nm) multilayer structures that undergo chemical modification without substantial material loss (Stuckey *et al.*, 2024 ▸). Complementary reflectometric and kinetic modelling studies have shown that these films undergo rapid oxidation by OH radicals with half-lives of minutes to an hour, impacting aerosol optical properties and potentially reducing planetary albedo (Shepherd *et al.*, 2025 ▸). Together, these studies underscore the unique ability of NR to provide depth-resolved non-destructive information that links nanoscale interfacial chemistry to macroscopic outcomes in clean energy materials, atmospheric reactivity and climate-relevant processes.

In summary, the research examples discussed here demonstrate that NR, using both unpolarized and polarized neutrons, offers exceptional potential for addressing a wide range of pressing scientific challenges – from ecology and cell membranes to superfluidity and hydrogen sensing. The instrumentation of high-flux TOF reflectometers at ISIS continues to advance in terms of neutronics and sample environments, providing researchers worldwide with ever-greater experimental capabilities.

## 5. Conclusions

The ISIS Neutron and Muon Source hosts a comprehensive suite of world-class neutron reflectometers, each tailored to specific scientific challenges while collectively covering a wide range of sample types, measurement geometries and experimental conditions. Over decades, neutron reflectometry has evolved into a robust, non-destructive and highly versatile technique, capable of probing buried interfaces with sub-nanometre resolution and delivering both structural and kinetic information under realistic sample environments. The ISIS reflectometers – SURF, INTER, POLREF and OFFSPEC – leverage high-flux pulsed neutron beams and advanced instrumentation to enable studies across soft matter, magnetic systems, quantum fluids and functional interfaces, providing access to horizontal and vertical scattering geometries, polarized and unpolarized measurements, and flexible resolution settings for specular, off-specular and grazing-incidence small-angle scattering.

The combination of versatile sample environments, including controlled temperature, pressure and magnetic field, with advanced data acquisition and analysis platforms ensures that experiments can be conducted under tightly controlled conditions while facilitating efficient data reduction, fitting and modelling. The TOF technique, position-sensitive detectors and multi-axis sample stages allow simultaneous acquisition of specular and off-specular scattering, supporting emerging methodologies such as PNR, PA and π-GISANS.

Recent scientific highlights demonstrate the broad applicability and capabilities of NR at ISIS. Collectively, these capabilities position ISIS neutron reflectometers as indispensable tools for investigating the structure and dynamics of complex interfaces across a broad spectrum of disciplines. The continued development of instrumentation, sample environments and computational resources ensures that NR at ISIS remains at the forefront of experimental research, supporting both fundamental scientific discovery and applied technological innovation.

## Figures and Tables

**Table 1:** Key parameters of the ISIS reflectometers

Instrument	Incident wavelength (Å)/moderator	Incident angle (°)/detector angle (°)†	Sample orientation	*L*_1_ (m)/*L*_2_ (m)‡	*Q_z_* range (Å^−1^)	Resolution, Δ*Q_z_*/*Q_z_*	Detector	Neutron flux at sample (cm^−2^ s^−1^)§
OFFSPEC	1.0–14.5	2.3	Horizontal	23.7	0.003–0.9	0.005–0.04	1D PSD (shifting fibre linear scintillator)	1 × 10^7^
	Cold grooved H_2_ moderator	0–8		3.3				

POLREF	1.0–15	2.3	Horizontal & vertical	23	0.006–0.8	0.01–0.05	1D PSD (OSMOND, microstrip gas chamber)	1 × 10^7^ (NR)
	Cold grooved H_2_ moderator	0–8 (H)		3				5 × 10^6^ (PNR)
		0–12 (V)						

INTER	1.3–17	2.3	Horizontal	17	0.006–0.8	0.01–0.05	Pseudo-2D PSD (shifting fibre multi-detector)	4 × 10^7^
	Cold grooved H_2_ moderator	0–9		3				

SURF	0.5–7	1.5	Horizontal	9	0.005−1.1	0.01–0.05	0D (single He^3^ gas detector)	5 × 10^6^
	Cold H_2_ moderator	0–10		2				

† Incident angle – initial inclination of a beamline relative to horizon; detector angle – 2θ angle between incident and reflected beams.

‡ *L*_1_ and *L*_2_ are source–sample and sample–detector distances, correspondingly.

§ Measured at typical slit gap values and normalized to the beam cross-sectional area. Corresponding wavelength distributions are shown in Fig. 3 ▸.

**Table 2:** Availability of experimental techniques

Instrument	Specular reflectometry	Off-specular scattering	π-GISANS	Isotopic contrast variation	Magnetic layer contrast variation	Polarization analysis	Free liquid surfaces
OFFSPEC	Yes	Yes	No	Yes	No	No	No
Multipurpose high-resolution reflectometer							

POLREF	Yes	Yes	No	Yes	Yes	Yes	No
Polarized reflectometer							

INTER	Yes	Yes	Yes	Yes	No	No	Yes
Flexible high-flux reflectometer							

SURF	Yes	No	No	Yes	No	No	Yes
Liquid reflectometer							

**Table 3:** Sample environment equipment† relevant to neutron reflectometry

Sample environment item		Typical parameters‡		Application notes
Solid–liquid cells	Peltier controlled cells	5–60°C	3 ml	Low-background solid/liquid interface studies (wetting, adsorption, membranes).
	Cells with liquid heating/cooling	0–80°C	3 ml	
	Magnetic contrast SAH type solid/liquid cell	0–80°C	3 ml	
	Electrochemical cells	Electrochemical potential control; moderate heating		*In situ* studies of electrochemical interfaces, battery films, electrodeposition on planar electrodes.

Liquid troughs	7 position liquid troughs	RT–60°C		Free liquid surfaces’ physical chemistry, studies of monolayers and floating films at the air–liquid interface, atmospheric studies.
	5 position liquid troughs	RT–60°C		
	NIMA Langmuir troughs	0–60°C; surface pressure control, various sizes		Allows monolayer compression; isotherms plus reflectivity of Langmuir films.
	Flow trough	Variable flow rates		Possibility of automated subphase exchange without disturbing the surface layer.

Humidity chambers	Salt-controlled box	0–95% RH; moderate heating		Studying polymer swelling, biomaterials and responsive coatings.
	Water-flow-controlled chamber	0–95% RH; moderate heating		

Heating	Water baths	0–90°C		Moderate temperature control.
	Hotplates	20−200°C; 1- or 4-position		Controlled heating for film annealing and temperature ramps with fast response.
	High-temperature furnace	20–350°C; sample is run in vacuum		High-*T* phase behaviour. Compatible with GMW magnet.
	Vacuum cells	RT–200°C		Research in vacuum or gas environments. Optical windows for Vis–UV irradiation.

Below/above room temperature	1-position Linkam chamber	−196–300°C		Phase diagram research at both negative and positive temperatures.

Cryostats	He flow (little blue)	2.8–300 K		Low-*T* studies of superconductors, magnetic thin films, temperature-dependent structural transitions. Often used with magnets or vacuum sample chambers.
	Helium reservoir cryostat (Orange)	1.4–300 K		
	Variox (big blue)	1.5–300 K		

Magnets	GMW 3473	0–2.5 T; pole gap 96 mm		Magnetic reflectometry, field-dependent phase transitions. Split-pair magnets allow horizontal sample plane for liquid surfaces. Often used in combination with cryostat for PNR with temperature control.
	GMW 5403	0–2 T; pole gap 38 mm		
	High-temperature superconductor	0–3 T; pole gap 80 mm		Room-temperature magnetic reflectometry.
	3-axis magnet (Helmholtz type)	0–2 T rotatable field; pole gap 70 mm		Studies requiring field direction control (in-plane versus out-of-plane magnetization) or complex magnetization states.

Accessories	High pressure liquid chromatography pumps	4-channel		Mixing and delivering liquids to cells.
	Switch valves	6-channel		
	Syringe pumps	Continuous cycle		

Inline sample characterization	QSense E4 QCM-D (quartz crystal microbalance with dissipation monitoring)	4 sensors, 1– 72 MHz, 7 harmonics		Real-time changes in mass and viscoelastic properties of films and surfaces.
	Densitometers	0.5–2 (±10^−4^) g m^−3^		Density measurements by a digital oscillating U-tube.
	Infrared reflection–absorption spectroscopy	4000–400 cm^−1^		Analysis of the molecular structure and orientation of thin films on reflective surfaces.

Offline sample preparation tools	Dipping troughs	Langmuir–Blodgett film depositions		Sample deposition on flat substrates.
		Langmuir–Schaeffer dipping		
	Spin-coater	1000–6000 rpm		

† Details can be found on the official ISIS source page (https://www.isis.stfc.ac.uk).

‡ RT: room temperature.

## Data Availability

The datasets analysed during the current study are available from the corresponding author on reasonable request.
